# Morphology and Molecular Analysis of *Moesziomyces antarcticus* Isolated From the Blood Samples of a Chinese Patient

**DOI:** 10.3389/fmicb.2019.00254

**Published:** 2019-02-15

**Authors:** Yuan Liu, Ziying Zou, Zonghai Hu, Wenbo Wang, Jie Xiong

**Affiliations:** ^1^Clinical Laboratory, The General Hospital of Western Theater Command, Chengdu, China; ^2^Centers for Disease Control and Prevention, Western Theater Command, Chengdu, China

**Keywords:** *Moesziomyces*, taxonomy, *Moesziomyces antarcticus*, fungemia, morphology, internal transcribed spacer

## Abstract

**Objective:** To identify the pathogen causing fungemia in a Chinese patient and describe its morphological and molecular characterizes.

**Methods:** Samples of central and peripheral venous blood were collected for blood culture. Morphology and drug sensitivities of the isolated yeast-like fungus were analyzed. rDNA sequencing and molecular phylogenetic analysis of the isolated strains were performed using DNAMAN and MEGA software.

**Results:** A strain of yeast-like fungi was repeatedly isolated from blood samples of a Chinese patient. The isolates grew well on sabouraud medium broth plate. The colonies were smooth and round at 28°C, and were of rough surface and irregular shape at 35°C. Molecular phylogenetic trees constructed based on the internal transcribed spacer (ITS) and D1/D2 domains of 28S rDNA gene demonstrated the isolated yeast-like fungus was *Moesziomyces antarcticus*. Drug susceptibility test showed that this isolated *M. antarcticus* was resistant or had relatively low susceptibility to flucytosine, fluconazole, voriconazole, and itraconazole, and only sensitive to amphotericin.

**Conclusion:** This study provided more information for the molecular and morphology characteristics of *M. antarcticus* and reviewed the species information of *Moesziomyces* associated with human infections, which will contribute to the identification and diagnosis of *Moesziomyces* infections.

## Introduction

The basidiomycetous yeast *Moesziomyces antarcticus* (previously cited as *Pseudozyma antarctica*), is originally isolated from a sediment sample obtained from Lake Vanda in Antarctica (Sugiyama et al., 1967), and belongs to the order *Ustilaginales* (*Ustilaginomycetes* and *Ustilaginomycotina*). *Moesziomyces* spp. is mainly isolated from plant surfaces and provides a natural source of protection against powdery mildews. Several *Moesziomyces* species have been reported to exhibit biological activity against biodegradable plastics, which are usually used in a number of industrial processes (Kitamoto et al., 2018). In recent years, more and more human cases infected by plant fungus have been reported. A total of 16 human cases caused by 10 kinds of *Moesziomyces* or *Pseudozyma* species had been reported in United States, China, Thailand, Brazil, India, France, Argentina, Korea and Nigeria from 2003 to 2015 (Table 1). Most of the cases were shown as fungaemia. Two dead cases were reported. One is a 78-year-old male patient diagnosed with astrocytoma in Korea, and a *Pseudozyma* species was isolated from the brain abscess (Hwang et al., 2010). Another dead case is a preterm low-birth-weight infant in Nigeria, and a strain of *M. bullatus* was isolated form her blood sample (Ojogba et al., 2015).

**Table 1:** Cases and strains information of *Moesziomyces* or *Pseudozyma* species infections in human.

No.	strains	Age/sex		Underlying condition			Clinical presentation		
*1*	*M. antarcticus*	N/A		N/A			N/A		
*2*	*M. antarcticus*	93/M		Hypertension, chronic renal insufficiency, Alzheimer’s disease, cerebral infarction			COPD		
*3*	*M. parantarctica*	N/A		N/A			N/A		
*4*	*P. thailandica*	N/A		N/A			N/A		
*5*	*M. aphidis*	7/F		Short gut syndrome			Fever, chill, malaise, and fatigue		
*6*	*M. aphidis*	51/M		Farmer, chronic leg swelling			Chronic mycetoma of leg		
*7*	*M. aphidis*	17/M		Burkitt lymphoma, chemotherapy			Neutropenic fever, lung infiltrates		
*8*	*M. aphidis*	0/M		Hemolytic jaundice			Lethargy, poor feeding		
*9*	*M. aphidis*	68/F		Adenocarcinoma of ampulla of Vater			Fever, chill		
*10*	*M. aphidis*	6/F		Osteosarcoma with lung metastasis			Neutropenic fever		
*11*	*M. aphidis*	51/M		AML, reinduction chemotherapy			Neutropenic fever, lung infiltrates		
*12*	*P. alboarmeniaca*	N/A		N/A			N/A		
*13*	*P. crassa*	N/A		N/A			N/A		
*14*	*P. siamensis*	N/A		N/A			N/A		
*15*	*Pseudozyma* spp.	78/M		Astrocytoma			Fever after brain surgery		
*16*	*Pseudozyma* spp.	52/F		Crohn’s disease, total colectomy state			Fever, headache, and weakness		
*17*	*M. bullatus*	0/F		preterm low birth weight			Low body temperature, jaundice, bilateral pitting pedal oedema		

**No.**	**Isolated specimen**	**MIC**						**Treatment**			**Country, year (reference)**
		**FLC**	**ITC**	**VRC**	**AMB**	**CAS**	**5FC**				

* 1*	Blood	0.5	0.06	N/A	0.12	N/A	>64	N/A		Thai, 2003 (Sugita et al., 2003)	
*2*	Blood	128	4	8	<0.5	N/A	>16	CAS→LAMB		**China, 2018 (This case)**	
*3*	Blood	2	0.25	N/A	0.25	N/A	>64	N/A		Thai, 2003 (Sugita et al., 2003)	
*4*	Blood	>64	>8	N/A	0.25	N/A	>64	N/A		Thai, 2003 (Sugita et al., 2003)	
*5*	Blood	4	0.12	N/A	0.25	N/A	N/A	FLC→ITC		United States, 2007 (Lin et al., 2008)	
*6*	Leg	N/A	N/A	N/A	N/A	N/A	N/A	ITC		China, 2011 (Chen et al., 2011)	
*7*	Pleural fluid	4	0.25	0.03	0.25	4	N/A	LAMB→VRC		Brazil, 2012 (Parahym et al., 2013)	
*8*	Blood	8	0.03	0.06	0.03	8	>64	AMB→VRC		India, 2013 (Prakash et al., 2014)	
*9*	Blood	16	0.19	0.03	0.19	>32	>32	LAMB		France, 2015 (Herb et al., 2015)	
*10*	Blood	2	0.03	0.03	0.13	N/A	128	LAMB		Argentina, 2013 (Orecchini et al., 2015)	
*11*	Blood	N/A	N/A	N/A	N/A	N/A	N/A	LAMB→VRC		Korea 2015 (Joo et al., 2016)	
*12*	Blood	32	4	2	0.25	>16	>64	N/A		Thai, 2014 (Mekha et al., 2014)	
*13*	Blood	>64	4	2	0.25	>16	>64	N/A		Thai, 2014 (Mekha et al., 2014)	
*14*	Blood	32	4	2	0.12	>16	>64	N/A		Thai, 2014 (Mekha et al., 2014)	
*15*	Abscess of brain	N/A	N/A	N/A	N/A	N/A	N/A	N/A		Korea, 2009 (Hwang et al., 2010)	
*16*	Blood	N/A	N/A	N/A	N/A	N/A	N/A	FLC→VRC		United States, 2014 (Siddiqui et al., 2014)	
*17*	Blood	128	0.12	0.03	1	8	64	FLC		Nigeria, 2014 (Ojogba et al., 2015)	


*FLC, fluconazole; ITC, itraconazole; VRC, voriconazole; AMB, amphotericin B; CAS, caspofungin; 5FC, flucytosine; LAMB, liposomal amphotericin B; N/A, not available.*

Only one case was infected with *M. antarcticus* (*Pseudozyma Antarctica* used in the report) in 2003, Thailand, while case information were not shown in this report (Sugita et al., 2003). In this study, we isolated a strain of *M. antarcticus* from the blood of a Chinese patient, which is the second reported case infected by *M. antarcticus* to date in the world. Here we present the isolation and morphology of the yeast-like genus *M. antarcticus.*

## Materials and Methods

### Sequence Analysis

In Feb and Mar 2018, a strain of yeast-like fungus (sample No. 1801245875) was isolated from blood samples of a patient in General Hospital of Chengdu Military Region. Nuclear DNA from these isolates was extracted. The D1/D2 regions of 28S rDNA (also as ribosomal large subunit, LSU), the ribosomal small subunit (SSU) sequence of 18S rDNA and the internal transcribed spacer (ITS) regions of the rRNA gene were sequenced directly from PCR products using the primers 5.8SR/LR7, SR1R/SR6, and ITS-1/ITS-4, respectively (Guzmán-Dávalos et al., 2017). This strain has been deposited in China Centre for Type Culture Collection (CCTCC)^1^.

The nucleotide sequences determined in this study have been deposited with GenBank and the accession numbers were MH185803 for ITS, MH185804 for LSU and MH185805 for SSU. Alignment of sequences was performed using DNAMAN software (Lynnon Biosoft). Phylogenetic trees were constructed using MEGA version 6.06 by neighbor-joining method with 1000 bootstrap replicates.

### Morphological and Physiological Characteristics

The isolated fungus was inoculated on Shalegar medium and cultured at 28 and 35°C. The colony growth was observed each day. The morphological characteristics were examined using a DM2000 microscope system (Leica, Germany).

### *In vitro* Susceptibility Test

The MIC of 5-flucytosine (5-FC), fluconazole, voriconazole, amphotericin B, and itraconazole was determined using the ATB FUNGUS 3 (bioMérieux, La Balme-les Grottes, France) in accordance with the manufacturer’s instructions. The microplates were incubated at 35°C for 48 and 72 h.

## Results

The patient is a 93-year-old male patient, who had chronic obstructive pulmonary disease (COPD) carrying a tracheal intubation after tracheostomy more than 1 year ago. In addition the patient was underlying causes of hypertension, chronic renal insufficiency, Alzheimer’s disease, cerebral infarction. On hospital day 9, fever developed as high as 38.5°C. Central and peripheral venous blood samples were collected for microbial examination and blood culture test, which showed a yeast-like fungus after cultured 12 to 24 h. Drug susceptibility test showed that the isolated fungus was resistant or had relatively low susceptibility to flucytosine, fluconazole, voriconazole and itraconazole, and only sensitive to amphotericin (Supplementary Table S2). Considering the nephrotoxicity of liposomal amphotericin B (LAMB), the patient was then treated with caspofungin. Central and peripheral venous blood samples were collected for culture at 3 and 6 days after caspofungin treatment. Fungemia was not under control, and caspofungin was changed to LAMB. After 2 weeks of treatment, the results of blood culture tests were all fungus negative.

**FIGURE 1:**
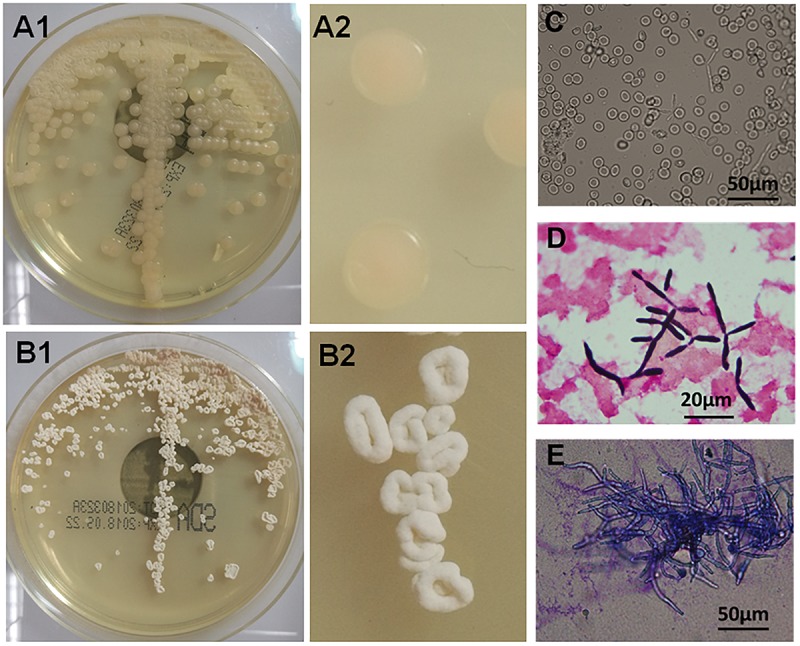
Morphological and physiological characteristics of isolated *Moesziomyces antarcticus*. **(A1,A2)** Smooth colonies of *M. antarcticus* after 2 days growth on sabouraud medium at 28°C. **(B1,B2)** Colony morphology of *M. antarcticus* after 2 days growth on sabouraud medium at 35°C. Direct microscopic examination **(C)** and gram staining **(D)** of fungi in blood culture flasks. **(E)** The hyphae cells were examined with methylene blue staining after growing in sabouraud medium broth at 35°C for 4 days.

The yeast-like fungus grew well on sabouraud medium broth, and had a different colony features cultured at different temperature. The colonies were smooth and round when cultured at 28°C (Figure 1A1,A2), and were of rough surface and irregular shape cultured at 35°C (Figure 1B1,B2). The isolated strains had a yeast-like form culture in the blood culture flasks (Figure 1C) and synnema-like structure (Figure 1D) on sabouraud medium, and could also form long tubular septate hyphae (Figure 1E). The fungus exhibited as lavender colored yeast-like colonies on the chromogenic agar medium of CHROMagar (Supplementary Figure S1). It is difficult to identify the species of the pathogen from the morphological and physiological characterizes. For molecular identification, the ITS sequence of isolated fungus showed 100% identity to *P. antarctica* (GenBank accession No. AB089358), and had one site of mutation of C-A compared with *P. antarctica* strains (GenBank accession Nos. AF294698, JN942669, and JX094775). Molecular phylogenetic trees constructed based on ITS and D1D2 demonstrated the isolated yeast-like fungus exhibited the highest sequence similarity with *P. antarctica* (Supplementary Figures S2, S3). As mentioned above and in discussion section, *P. antarctica* was now renamed to *M. antarcticus*.

*Moesziomyces* infected case in China is rare. There has been only one reported human infection caused by *M. aphidis* in mycetoma on the leg, while this isolation is not available for us. Several related *Moesziomyces* species were obtained from China General Microbiological Culture Collection Center (Supplementary Table S1). All the tested *Moesziomyces* species had similar spore and mycelium characterizes. It was interesting to note that only *M. antarcticus* exhibit biphasic growth characteristics at 28 and 35 degree. Morphology characterizes of colonies of *M. tsukubaensis*, *M. hubeiensis*, *M. aphidis*, and *M. rugulosus* were extremely similar growth at 28 and 35 degree (Supplementary Figure S1). Drug susceptibility test showed that *M. antarcticus* and *M. rugulosus* were only sensitive to AMB, while *M. tsukubaensis*, *M. hubeiensis* and *M. aphidis* were sensitive to AMB, VRC, and ITC (Supplementary Table S2).

## Discussion

Taxonomy of fungi has always been a tricky puzzle to microbiologist. *M. antarcticus* was initially described as *Sporobolomyces antarcticus* isolated from the 9-m deep sediment of Lake Vanda in Antarctica (Goto et al., 1969), which was transferred to the genera *Candida* in 1983 (Barnett et al., 1983) and *Vanrija* in 1987 (Moore, 1987). Then the species was combined with the genus *Pseudozyma* and described as *Pseudozyma antarctica* in 1995 (Boekhout, 1995). Recently, *P. antarctica* was transferred to the genus *Moesziomyces* in 2015 and now described as *M. antarcticus* (Wang et al., 2015; Kruse et al., 2017).

*Moesziomyces* infections in humans have been repeatedly reported since the first description as a human pathogen in 2003. Most of these cases were immunocompromised patients and carried out foreign materials, such as central venous catheter. In this study, we reported a strain of *M. antarcticus* isolated from the blood samples of a Chinese patient. The patient was ‘super senior’ elderly and had multiple chronic conditions who belonged to immunocompromised person. The case stayed in bed for a long period of time, and multiple catheters were placed into his body including a central vein catheter, an urinary catheter an airway catheter. The catheters may be one of the causes of fungal infection in this case.

A total of 10 species of *Moesziomyces* were reported in 17 human cases (Table 1), among which *M. aphidis* is most frequent (7/17). Over 80% (14/17) of these strains were isolated from blood samples, the other three were isolated form leg, pleural fluid and abscess of brain, respectively. Morphological characterizes of these isolates were similar between each other, which made it difficult to identify the species from morphology. Drug susceptibility of these isolates was quite different. *M. antarcticus* isolated by Sugita in Thailand, in 2003 was sensitive to fluconazole (FLC) and itraconazole (ITC), while our isolate in this case was resistant to FLC and ITC. The intensive use of antimicrobial drugs probably increased the microbial resistance.

It is worth to note that rare fungi have recently been implicated in human infections and have increased dramatically over recent decades especially in immunocompromised patients. This may be due to the continuous expansion of the field of human behaviors and the other due to the advances in diagnostic techniques. The risk factors included immunosuppression and foreign material, such as catheter. Molecular analysis of rDNA sequences is still an effective tool for fungi identification.

## Ethics Statement

This study was conducted according to the principles of the Declaration of Helsinki and approved by the ethics committee of General Hospital of Chengdu Military Region. This study aims to analysis the morphology and molecular analysis of the isolated pathogens and does not involve patients’ identification information. Therefore, the need for written informed consent was waived and oral informed consent was obtained.

## Author Contributions

YL and JX conceived and designed the experiments and wrote the paper. YL and ZZ carried out the DNA extraction and library preparation. ZH and WW contributed to sequencing and phylogenetic analysis. All authors read and approved the final manuscript.

## Conflict of Interest Statement

The authors declare that the research was conducted in the absence of any commercial or financial relationships that could be construed as a potential conflict of interest.

## Footnotes

1 http://www.cctcc.org
