# Supplementary material for: Morphology and Molecular Analysis of Moesziomyces antarcticus Isolated From the Blood Samples of a Chinese Patient
Source: Front Microbiol. 2019 Feb 15;10:254. doi: 10.3389/fmicb.2019.00254 (PMC6384246; doi:10.3389/fmicb.2019.00254)
Supplement: Supplementary file 1 [file Data_Sheet_1.docx]

Table S1.Strain information of *Moesziomyces* or *Pseudozyma* species used in this study

| Species | CGMCC No. | Source | Date | Location | Country |
| --- | --- | --- | --- | --- | --- |
| *M. antarcticus* | 2.3605 | Plant | 5/22/2007 | Hainan | China |
| *P. tsukubaensis* | 2.4039 | Apple peel | 1/7/2009 | Beijing | China |
| *P. hubeiensis* | 2.3590 | Plant | 5/22/2007 | Hainan | China |
| *M. aphidis* | 2.4679 | Plant leaf | 12/24/2012 | Jiangxi | China |
| *M. rugulosus* | 2.3301 | Banana | 12/11/2006 | Yunnan | China |


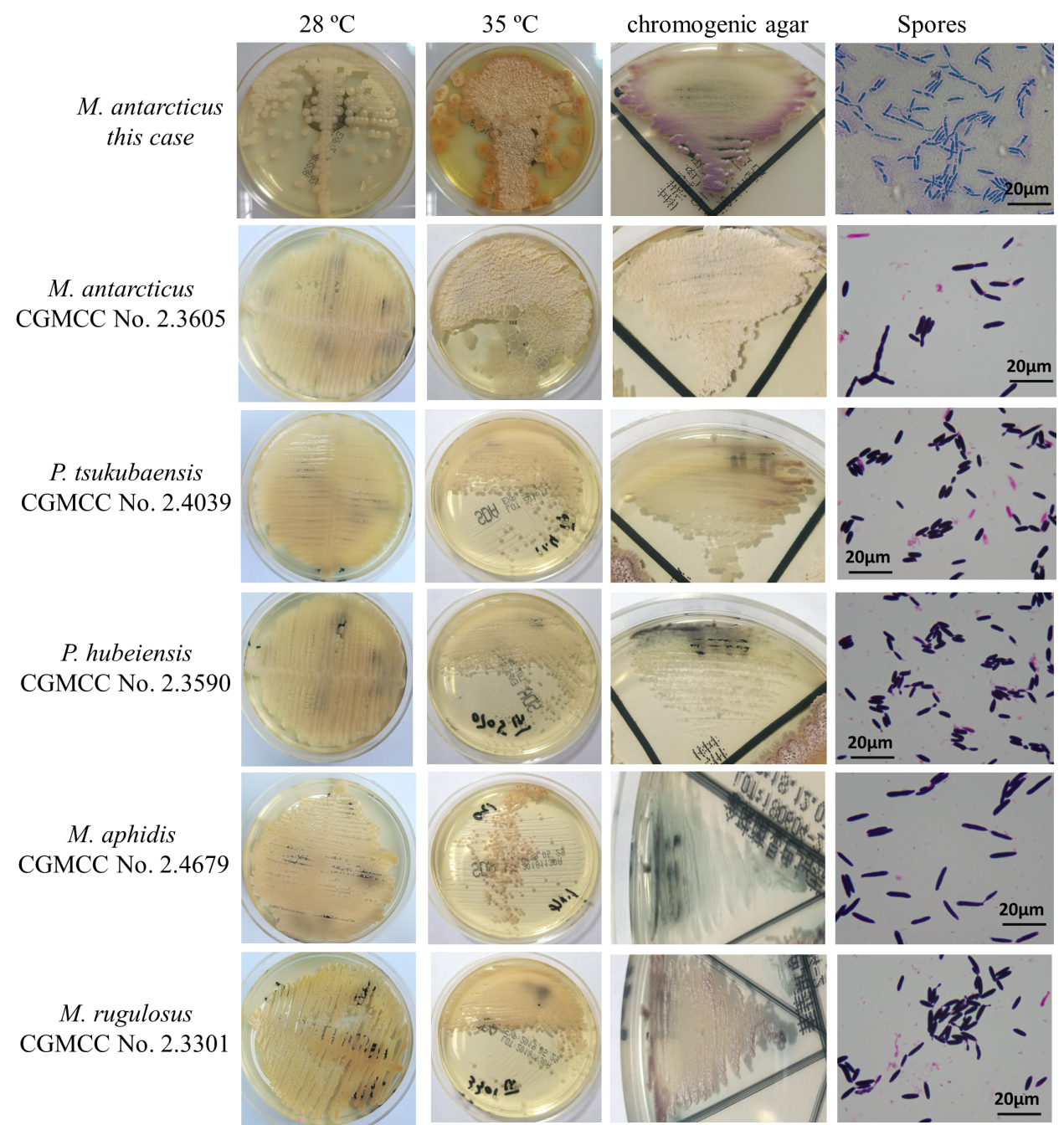


**Figure S1. Morphology characterizes of *Moesziomyces* species obtained from CGMCC.** *M. antarcticus* exhibit biphasic growth characteristics at 28 and 35℃. Morphology characterizes of colonies of *M. tsukubaensis*, *M. hubeiensis*, *M. aphidis* and *M. rugulosus* were extremely similar growth at 28 and 35 ℃. *M. antarcticus* isolated in this case, *M. rugulosus* (CGMCC No. 2.3301) and *M. tsukubaensis* (CGMCC No. 2.4039) exhibited as lavender colored yeast-like colonies on the chromogenic agar medium of CHROMagar. *M. antarcticus* (CGMCC No. 2.3605) showed as white colonies. *M. hubeiensis* (CGMCC No. 2.3590) and *M. aphidis* (CGMCC No. 2.4679) exhibited as blue colored colonies on CHROMagar.

Table S2. In vitro susceptibility of *Moesziomyces* or *Pseudozyma* species to antifungal agents

| Species | CGMCC No. | MIC (mg/mL) | | | | |
| --- | --- | --- | --- | --- | --- | --- |
|  |  | 5-FC | FLC | VRC | AMB | ITC |
| *M. antarcticus* | This case | >16 | >128 | >8 | <0.5 | >4 |
| *M. antarcticus* | 2.3605 | 16 | 128 | 8 | <0.5 | 4 |
| *P. tsukubaensis* | 2.4039 | 16 | 16 | 0.5 | <0.5 | 0.25 |
| *P. hubeiensis* | 2.3590 | 16 | 4 | 0.125 | <0.5 | <0.125 |
| *M. aphidis* | 2.4679 | 16 | 4 | 0.25 | <0.5 | 0.5 |
| *M. rugulosus* | 2.3301 | 16 | >128 | 1 | <0.5 | >4 |

5-FC, flucytosine; FLC, fluconazole; VRC, voriconazole; AMB, amphotericin B; ITC, itraconazole.

*Pseudozyma antarctica* (JN942669)

*Pseudozyma antarctica* (JX094775)

*Pseudozyma antarctica* (AF294698)

**1801245875 (MH185803)** O

*Pseudozyma antarctica* (AB089358)

*Moesziomyces antarcticus* (MG871193)

*Pseudozyma* sp. SMN01 (KF922220)

*Pseudozyma rugulosa* (AB089370)

*Pseudozyma aphidis* (JQ425372)

*Pseudozyma* sp. HB 1191 (AM160634)

*Pseudozyma* sp. HB 1203 (AM160637)

*Pseudozyma aphidis* (AB089362)

*Metschnikowia* sp. JSKim-2014 (AB998371)

*Pseudozyma aphidis* (FN424100)

*Pseudozyma parantarctica* (AB089356)

*Moesziomyces bullatus* (DQ831013)

*Pseudozyma thailandica* (AB089354)

*Pseudozyma tsukubaensis* (AB089372)

*Pseudozyma* sp. DMST 17137 (AB117963)

*Ustilago hordei* (AY345003)

*Ustilago calamagrostidis* (AY740065)

*Pseudozyma shanxiensis* (DQ008956)

*Pseudozyma fusiformata* (AB089366)

*Pseudozyma hubeiensis* (DQ008954)

*Sporisorium holwayi* (AY344980)

*Sporisorium sorghi* (AY740021)

99

94

98

52

100

68

94

97

94

100

50

67

54

63

86

98

66

77

52

53

0.01

**Figure S2. Molecular phylogenetic trees constructed using the ITS sequence of the isolated strain and related ustilaginomycetous anamorphic yeasts.** The GenBank accession numbers are indicated in parentheses. The number at each branch points is the percentage supported by bootstrap. Bar: 1% Sequence divergence. O: The sequence obtained in this study. *Pseudozyma antarctica*, *P. aphidis*, *P. parantarctica* and *P. rugulosa* had been transferred to *Moesziomyces* (Wang et al., 2015).

*Moesziomyces antarcticus* (KY108571)

*Pseudozyma* sp. SMN01 (KF922220)

**1801245875 (MH185804)** O

*Pseudozyma* sp. VITJzN01 (JX454447)

*Ustilago maydis* (FJ644528)

*Kalmanozyma brasiliensis* (KF737866)

*Kalmanozyma brasiliensis* (XR 001630178)

*Pseudozyma hubeiensis* (XR 001099828)

*Ustilago maydis* (MH047195)

100

99

57

51

69

100

0.005

**Figure S3. Molecular phylogenetic trees constructed based on the LSU sequences of the isolated strain and related ustilaginomycetous anamorphic yeasts.** The GenBank accession numbers are indicated in parentheses. The number at each branch points is the percentage supported by bootstrap. Bar: 0.5% Sequence divergence. O: The sequence obtained in this study.
